# Updating the Species Diversity of Pestalotioid Fungi: Four New Species of *Neopestalotiopsis* and *Pestalotiopsis*

**DOI:** 10.3390/jof10070475

**Published:** 2024-07-11

**Authors:** Weishan Zhang, Yixuan Li, Lu Lin, Aoli Jia, Xinlei Fan

**Affiliations:** 1 The Key Laboratory of Efficient Production of Forest Resources, Beijing Forestry University, Beijing 100083, China; weishanzhang@bjfu.edu.cn (W.Z.); yixuanli@bjfu.edu.cn (Y.L.); lulin0677@bjfu.edu.cn (L.L.); nice2cu@bjfu.edu.cn (A.J.); 2 The Key Laboratory for Silviculture and Conservation of the Ministry of Education, Beijing Forestry University, Beijing 100083, China

**Keywords:** classification, identification, Pestalotiopsidaceae, phylogenetic analyses

## Abstract

Pestalotioid fungi are associated with a wide variety of plants around the world as pathogens, endophytes, and saprobes. In this study, diseased leaves and branches of plants were collected from Guizhou and Sichuan in China. Here, the fungal isolates were identified based on a phylogenetic analysis of the internal transcribed spacer region (ITS), the translation elongation factor 1-alpha (*tef1*-*α*) and the beta-tubulin (*tub2*) of ribosomal DNA, and the morphological characteristics. Ten *Neopestalotiopsis* isolates and two *Pestalotiopsis* isolates were obtained, and these isolates were further confirmed as four novel species (*N. acericola*, *N. cercidicola*, *N. phoenicis*, and *P. guiyangensis*) and one known species, *N. concentrica*.

## 1. Introduction

Coelomycetous fungi are a diverse group of asexually reproducing fungi that belong to the phylum Ascomycota [1]. This group includes a wide variety of fungi that can be found in different environments, including soil, plant surfaces, and even in association with other organisms [1]. Coelomycetous fungi are pathogens of a wide variety of plants, with types such as endophytes, saprophytes, and pathogenic fungi [2]. Concerning *Sporocadaceae*, as a member of coelomycetous fungi, a lot of research has been carried out on their taxonomy and pathology based on phylogeny and morphology, and pestalotioid species are some of the most common pathogenic genera [3]. Pestalotioid species are the main group of pathogenic fungi that cause leaf spot disease in plants, and they have been found to cause serious ecological problems [4,5,6,7,8,9,10,11,12].

Species of pestalotioid fungi have various ecological behaviors as plant pathogens, endophytes, or saprobes, and are widespread in temperate and tropical regions [13,14,15]. *Pestalotiopsis* was divided from *Pestalotia* as a distinct genus, on the basis of varying numbers of conidia, by Steyaert in 1949 [16]. Subsequently, Nag Raj [17,18] argued that the categorization of many species in *Pestalotiopsis*, as delineated by Steyaert, is problematic and pointed out that the type of species associated with *Pestalotiopsis* must be re-examined. In 2014, Maharachchikumbura et al. [15] reclassified *Pestalotiopsis* into three genera (*Neopestalotiopsis*, *Pestalotiopsis*, and *Pseudopestalotiopsis*). *Neopestalotiopsis* differs from *Pestalotiopsis* and *Pseudopestalotiopsis* based on its multicolored median cells [15]. *Pseudopestalotiopsis* can be distinguished from *Pestalotiopsis* by comparing the color of the concolorous median cells for those possessing equally pigmented median cells [15]. Currently, the three genera are grouped as Pestalotioid fungi [15].

**Figure 1:**
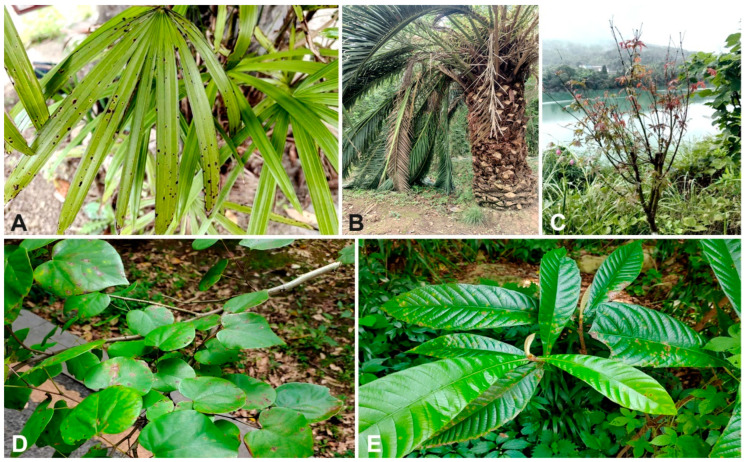
Diseased plants in Sichuan and Guizhou: (**A**) Symptoms of *Rhapis excelsa* in Sichuan; (**B**) Leaf spots of *Phoenix canariensis* in Sichuan; (**C**) Branch dieback of *Acer palmatum* in Sichuan; (**D**) Pathogenic fungi on *Cercis chinensis* leaves in Guizhou; (**E**) Leaf spots of *Eriobotrya japonica* in Guizhou.

Many novel species have been introduced into pestalotioid fungi in recent years [12]. Pestalotioid fungi primarily cause branch and leaf diseases, including canker lesions, leaf spots, gray blight, fruit rots, and various post-harvest diseases [12,15,19,20,21,22,23,24,25,26,27,28,29,30]. For example, *Neopestalotiopsis amomi* causes leaf blight in *Amomum villosum* [20]. Pestalotioid fungi were found to cause stem girdling and dieback in young eucalyptus plants in Portugal [28]. The above examples show that, in plants, pestalotioid fungi are widespread as phytopathogenic hosts. The aim of this study was to identify the pestalotioid fungi collected from plants in China (Figure 1) based on both their morphological characters and the combination of molecular phylogentic analyses of ITS, *tef1*-*α*, and *tub2*.

## 2. Materials and Methods

### 2.1. Sample Collection and Isolation

A survey of plants in the Guizhou and Sichuan provinces of China in 2023 resulted in the collection of 98 samples (20 branches and 25 leaves in Sichuan; 28 branches and 25 leaves in Guizhou) with different symptoms, including 12 samples (10 samples in Sicuan and 2 samples in Guizhou) with typical symptoms. The leaves were isolated using the tissue isolation method [31], the surfaces of the leaves were gently wiped clean with distilled water, and the leaf spots were cut into small pieces (0.1 × 0.2 cm) and sterilized (75% ethanol for 30 s, 5% sodium hypochlorite for 45 s, and rinsed with distilled water). After disinfection, the leaves were placed on dry sterile filter paper to absorb the moisture, and then the leaves were placed on potato dextrose agar (PDA; 200 g potatoes, 20 g dextrose, 20 g agar per liter) and incubated at 25 °C in the dark until spores germinated. Pure cultures were obtained by cutting off hyphal tips of single germinating conidia, transferring them to new PDA plates, and incubating them in the dark at 25 °C. The surface of the blade was sterilized, and the top layer of fruiting bodies on the diseased branches was scraped off. Then, the fruiting bodies were placed on PDA medium and incubated at 25 °C under dark conditions until the spores germinated. Individual germinated conidia were transferred to fresh PDA plates to obtain pure cultures. Herbarium materials were deposited at the Museum of the Beijing Forestry University (BJFC). The 12 cultures obtained in this study were deposited at the China Forestry Culture Collection Centre, Beijing, China (CFCC).

### 2.2. DNA Extraction, PCR Amplification, and Phylogenetic Analysis

Genomic DNA was extracted from mycelium on the PDA using the CTAB method [32]. The PCR mixture consisted of 10 μL TopTaq™ Master Mix, 6 μL nucleasefree H_2_O, 1 μL of each primer, and 2 μL DNA. The samples were made up to the final volume of 20 μL. The amplification of partial gene sequences was carried out using three DNA fragments (ITS, the translation elongation factor 1-alpha (*tef1-α*), and beta-tubulin (*tub2*)). ITS used primer sets ITS1/ITS4 [33], *tef1-α* used primer sets EF1-728F/EF1-1567R [34], and *tub2* u primer sets T1/Bt-2b [35]. The genes and PCR conditions used for the different genera are listed in Table 1. The DNA was sequenced by the SinoGenoMax Company Limited (Beijing, China). The forward and reverse reads were assembled using Seqman v. 7.1.0. MEGA6 was used to manually compare and check the sequences [36]. Ambiguous regions were excluded from the alignments. Phylogenetic analyses were analyzed with Maximum Likelihood analysis (ML) and Bayesian Inference (BI) analysis. Maximum Likelihood analysis (ML) was performed in PhyMLv.7.2.8 [37], and MrBayes v. 3.2.0 was used for Bayesian Inference (BI) analysis [38]. Maximum Likelihood analysis (ML) was performed using the GTR site substitution model, and branch support was assessed using the bootstrapping (BS) method with 1000 repetitions [39]. Bayesian Inference (BI) analysis was performed using the Markov chain Monte Carlo (MCMC) algorithm [39]. For *Neopestalotiopsis*, the GTR+I model, with a certain proportion of invariant sites, was chosen for ITS, and the HKY+G model with gamma distribution rate was chosen for *tef1-α* and *tub2*. The GTR+I+G model was chosen for the ITS of *Pestalotiopsis*, and the HKY+I+G model was chosen for *tef1-α* and *tub2*. Two MCMC chains were run for 1,000,000 generations, starting from the random tree, and the first 25% of the trees were discarded every 1000 generations as the posterior probabilities (BPP). The posterior probabilities (BPP) for each analysis were used to evaluate the remaining trees [39]. The resulting trees were observed in Figtree v. 1.3.1.

## 3. Results

### 3.1. Phylogenetic Analyses

**Figure 2:**
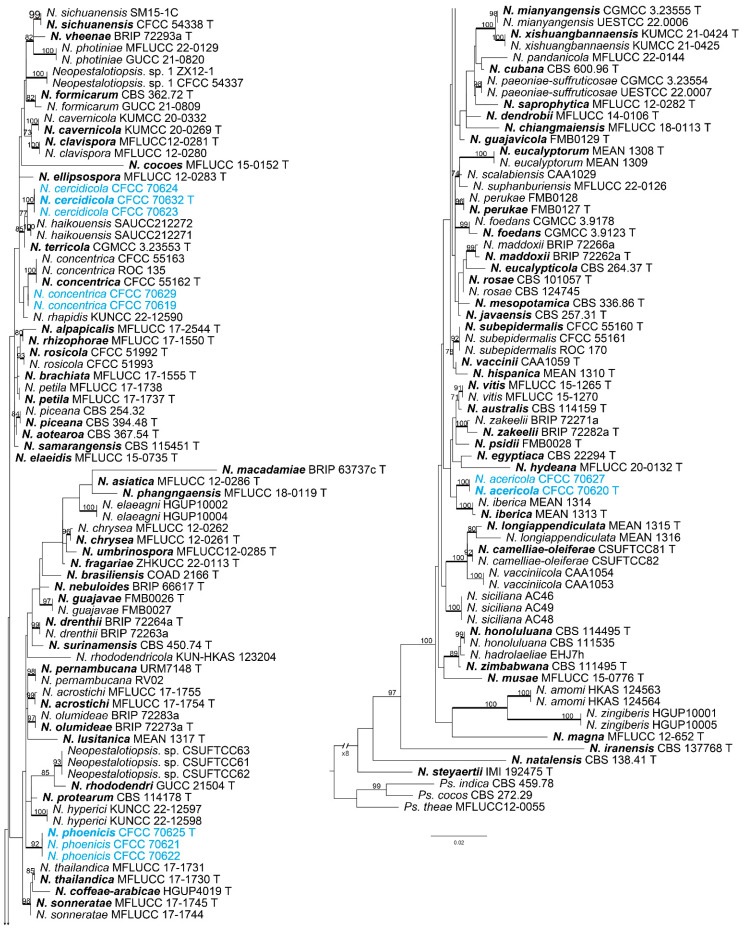
Phylograms were generated by maximum likelihood (ML) based on combined ITS, *tef1-α*, and *tub2* sequence data of *Neopetalotiopsis* isolates. The tree was rooted by *Ps*. *cocos* (CBS 272.29), *Ps*. *indica* (CBS 459.78), and *Ps*. *theae* (MFLUCC12–0055). Scale bars indicate 0.02 nucleotide changes per locus. ML bootstrap support values above 70% are shown near nodes. Thickened branches represent posterior probabilities above 0.95 from BI. Isolates from this study are marked in blue in the trees. Ex-type strains are in bold and labeled T.

**Figure 3:**
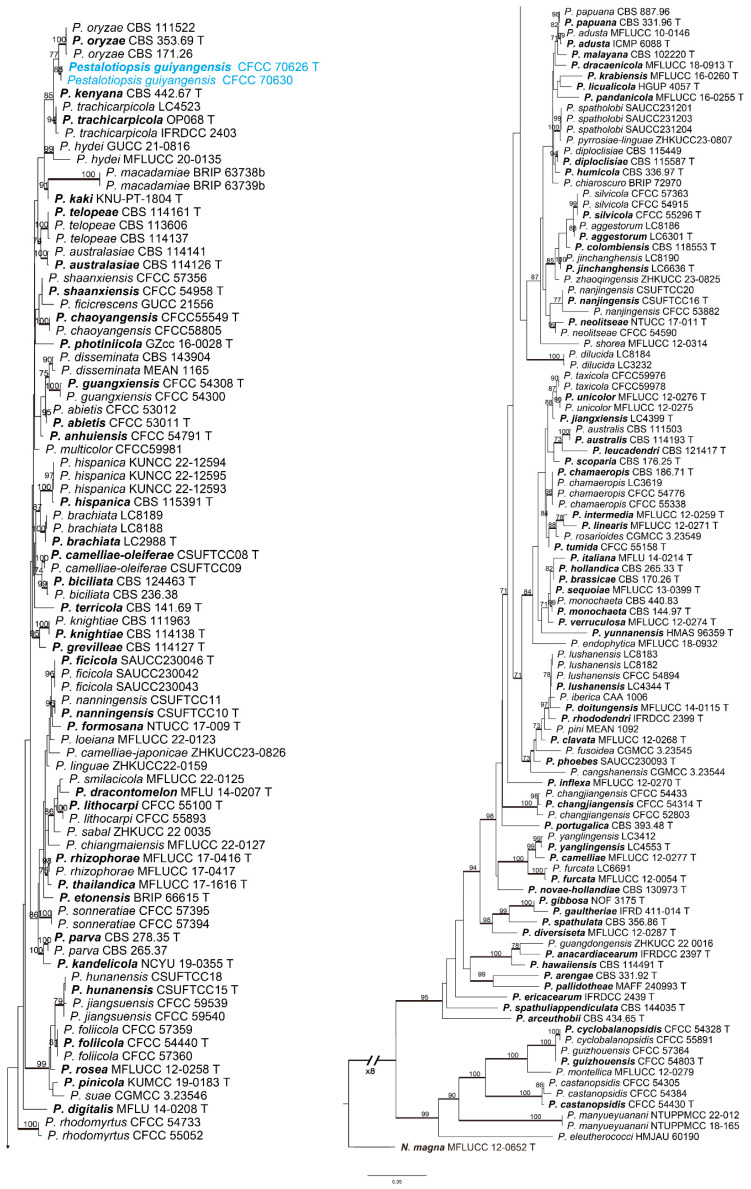
Phylograms were generated by maximum likelihood (ML) based on combined ITS, *tef1-α*, and *tub2* sequence data of *Pestalotiopsis* isolates. The tree was rooted by *N. magna* (MFLUCC 12–652). Scale bars indicate 0.05 nucleotide changes per locus. ML bootstrap support values above 70% are shown near nodes. Thickened branches represent posterior probabilities above 0.95 from BI. Isolates from this study are marked in blue in the trees. Ex-type strains are in bold and labeled T.

In this study, we combined the analysis of the concatenated DNA sequence datasets of ITS, *tef1-α*, and *tub2* to construct phylogenetic trees for *Neopestalotiopsis* and *Pestalotiopsis*. The combined species phylogeny of *Neopestalotiopsis* isolates consisted of 147 sequences, including outgroups *Pseudopestalotiopsis cocos* (culture CBS 272.29), *Ps*. *indica* (culture CBS 459.78), and *Ps*. *theae* (culture MFLUCC12–0055). A total of 2186 characters (556 in ITS, 882 in *tef1-α*, and 748 in *tub2*) were included in the phylogenetic analysis. The ML tree with bootstrap values and BI posterior probabilities is shown in Figure 2. The phylogenetic tree inferred from the concatenated alignment resolved the ten *Neopestalotiopsis* isolates into four well-supported monophyletic evolutionary branches representing three new and one known species of *Neopestalotiopsis*, respectively (Figure 2). In *Pestalotiopsis*, the phylogeny of the *Pestalotiopsis* isolates consisted of 192 sequences, including the outgroup *Neopestalotiopsis magna* (culture MFLUCC 12–652). A total of 1919 characters (536 in ITS, 572 in *tef1-α*, and 811 in *tub2*) were included in the phylogenetic analysis. Similar tree topologies were obtained using ML and BI methods (Figure 3). The two isolates of *Pestalotiopsis* decomposed into a well-supported monophyletic evolutionary branch representing a new species of *Pestalotiopsis* (Figure 3). All of the sequences obtained in this study were submitted to GenBank (Table S1).

### 3.2. Taxonomy

Five pestalotioid species were identified and characterized based on a polyphasic approach. There are three new species of *Neopestalotiopsis*, identified as *N. acericola*, *N. cercidicola*, and *N. phoenicis*, respectively, and one known species (*N. concentrica*). One *Pestalotiopsis* species was identified as *P. guiyangensis*. For all of these identified taxa, species descriptions and illustrations are given below.

**Figure 4:**
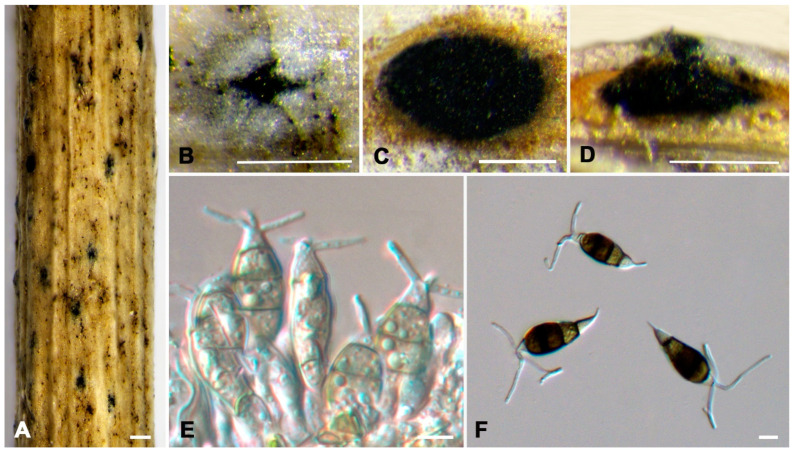
*Neopestalotiopsis acericola* (BJFC-S2333). (**A**,**B**) Habit of conidiomata on twig; (**C**) Transverse section of a conidioma; (**D**) Longitudinal section through a conidioma; (**E**) Conidiogenous cells giving rise to conidia; (**F**) Conidia. Scale bars: (**A**–**F**) = 10 μm.

***Neopestalotiopsis acericola*** W.S. Zhang & X.L. Fan, sp. nov. (Figure 4).

**MycoBank:** MB 854098

**Etymology:** Named after the genus of the host species, *Acer*.

**Holotype:** BJFC-S2333

**Description:** Conidiomata pycnidial in vivo. Asexual morph: Conidiomata ellipsoidal to rounded black and semi-immersed, beneath grayish, erumpent, and raised areas of the host epidermis, central black ostioles, 61–125 μm in diameter, rhombic to rounded, scattered or aggregate, with spores scattered around the dehisce with locules. Conidiophores reduced to conidiogenous cells, smooth and hyaline. Conidiogenous cells, ampulliform, discrete, thin-walled, smooth and hyaline. Conidia fusoid, ellipsoid to subcylindrical, straight or slightly curved, 19.0–24.0 × 6.5–8.0 μm (av. ± SD = 21.57 ± 1.17 × 7.53 ± 0.38 μm, n = 50), L/W = 2.4–3.3 μm (av. ± SD = 2.87 ± 0.21 μm, n = 50), 4-septate; basal cell conical, with a truncated base, hyaline or pale brown, thin-walled, and 3.0–6.0 μm long (av. ± SD = 4.45 ± 0.81 μm, n = 50); three median cells that are versicolored, minutely verruculose or smooth, septa and periclinal walls that are darker than the rest of the cell. The second cell from the base is honey brown and 3.0–6.0 μm long (av. ± SD = 4.31 ± 0.59 μm, n = 50); the third cell is dark brown and 4.0–6.5 μm long (av. ± SD = 5.19 ± 0.60 μm, n = 50); the fourth cell is brown and 3.5–5.5 μm long (av. ± SD = 4.55 ± 0.55 μm, n = 50); the apical cell is 2.5–4.5 μm long (av. ± SD = 3.32 ± 0.52 μm, n = 50), hyaline, inverted trapezoidal to conical, smooth, and thin-walled, with two or three tubular apical appendages arising from the apical crest, unbranched, filiform, and 15.0–24.5 μm long (av. ± SD = 20.55 ± 1.95 μm, n = 50); the basal appendage is single, unbranched, and 3.0–7.0 μm long (av. ± SD = 4.45 ± 0.81 μm, n = 50). A sexual morph was not observed.

**Culture characteristics:** The colonies on PDA reached up to 60 mm in diameter after seven days at 25 °C. The colonies were filamentous, with an undulate edge to circular, white, with a dense aerial mycelium on the surface, and the fruiting bodies were black.

**Typus:** CHINA, Sichuan Province, Guangyuan City, Lizhou District, 105°38′31″ E, 32°27′41″ N, from branches of *Acer palmatum*, Y.X. Li and L. Lin, 11 October 2023 (holotype BJFC-S2333, ex-holotype culture CFCC 70620); *ibid*. (paratype BJFC-S2334, ex-paratype culture CFCC 70627).

**Notes:** Two isolates from our collection developed an independent clade in the phylogenetic tree with 100% ML and 1.00 BI value (Figure 2). *N. acericola* was phylogenetically close to *N. iberica* (Figure 2), but there were eight nucleotide differences, including seven in *tef1-α* (427/435, 98.16%) and one in *tub2* (375/376, 99.73%) with *N. iberica*, and differed in conidia width (*N. acericola* (6.5–8.0 μm); *N. iberica* ((7.2) 8.2–8.7 (9.8) μm). Moreover, *N. acericola* had a smaller fourth cell (3.5–5.5 μm) than *N. iberica* (4.5–6.6 μm) and three median cells of *N. acericola* with minutely verruculose or smooth surface, three median cells of *N. iberica* with a smooth surface. Additionally, *Neopestalotiopsis acericola* was isolated from the branches of *Acer palmatum*. *Neopestalotiopsis iberica* was isolated from the leaves and stems of *Eucalyptus globulus* [28].

**Figure 5:**
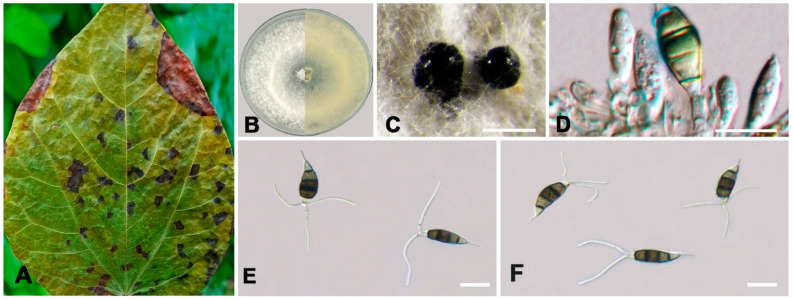
*Neopestalotiopsis cercidicola* (ex-holotype culture CFCC 70632). (**A**) Diseased leaf of *Cercis chinensis*; (**B**) Colony on PDA at seven days; (**C**) Conidial masses formed on PDA; (**D**) Conidiogenous cells; (**E**,**F**) Conidia. Scale bars: (**C**–**F**) = 10 μm.

***Neopestalotiopsis* *cercidicola*** W.S. Zhang & X.L. Fan, sp. nov. (Figure 5).

**MycoBank:** MB 854099

**Etymology:** Named after the genus of the host species, *Cercis*.

**Holotype:** BJFC-S2338

**Description:** Pathogenic on *Cercis chinensis* leaves. Asexual morph: Conidiomata pycnidial on PDA. Conidiomata are 200–300 μm in diameter, 47–148 μm high, globular, scattered or aggregated, and black. Conidiophores reduce to conidiogenous cells, smooth and hyaline. Conidiogenous cells are ampulliform, discrete, thin-walled, and smooth and hyaline. Conidia are shuttle-shaped, ellipsoid to subcylindrical, straight or slightly curved, smooth, 17.5–23.5 × 5.5–8.5 μm (av. ± SD = 20.71 ± 1.22 × 6.90 ± 0.62 μm, n = 50), L/W = 2.5–3.5 μm (av. ± SD = 3.02 ± 0.25 μm, n = 50), 4-septate; the basal cell is conical, with a truncated base, hyaline, smooth, thin-walled, and 2.5–5.0 μm long (av. ± SD = 3.60 ± 0.47 μm, n = 50); three median cells are versicolored, cylindrical, with the second cell from the base being pale brown and 3.0–5.0 μm long (av. ± SD = 4.20 ± 0.49 μm, n = 50); the third cell is honey brown and 3.5–5.5 μm long (av. ± SD = 4.84 ± 0.42 μm, n = 50); the fourth cell is pale brown and 3.5–5.0 μm long (av. ± SD = 4.36 ± 0.38 μm, n = 50); the apical cell is 2.0–4.0 μm long (av. ± SD = 3.26 ± 0.43 μm, n = 50), hyaline, inverted trapezoidal to conical, smooth, and thin-walled, with two or three tubular apical appendages arising from the apical crest, unbranched, filiform, and 15.0–27.5 (29.9) μm long (av. ± SD = 21.32 ± 3.54 μm, n = 50); a basal appendage is present and 3.0–5.5 μm long (av. ± SD = 4.26 ± 0.63 μm, n = 50). A sexual morph was not observed.

**Culture characteristics:** The colonies on PDA reached up to 59 mm in diameter after seven days at 25 °C. The colonies were filamentous to circular, white, with dense aerial mycelium on the surface, and the reverse color was pale yellow. Fruiting bodies were observed after seven days.

**Typus:** CHINA, Sichuan Province, Guangyuan City, Lizhou District, 105°51′23″ E, 32°25′04″ N, on leaf spots of *Cercis chinensis*, Y.X. Li and L. Lin, 9 October 2023 (holotype BJFC-S2338, ex-holotype culture CFCC 70632); *ibid*. (paratype BJFC-S2339, ex-paratype culture CFCC 70624).

**Additional material examined:** CHINA, Sichuan Province, Guangyuan City, Lizhou District, 105°51′23″ E, 32°25′04″ N, on leaf spots of *Cercis chinensis*, Y.X. Li and L.L, 9 October 2023 (BJFC-S2340, living culture CFCC 70623).

**Notes:** Phylogenetic analysis combining the DNA sequence datasets of ITS, *tef1-α*, and *tub2* revealed that *N. cercidicola* formed a separate branch (BI/ML = 1/100) (Figure 2). *N. cercidicola* was phylogenetically close to *N. haikouensis* (Figure 2), but there were five bp different in the concatenated alignment (one nucleotide difference in ITS, 484/485, 99.79%; two in *tef1-α*, 432/434, 99.53%; and two in *tub2*, 710/712, 99.71%) with *N. haikouensis*, and differed in host and culture characteristics (*N. cercidicola* from leaf spots of *Cercis chinensis*, colonies filamentous to circular, white, with dense aerial mycelium on the surface, and the reverse color was pale yellow; *N. haikouensis* from leaf spots of *Ilex chinensis*, colonies edge undulate, white to gray-white, with moderate aerial mycelium on the surface, and the reverse was similar in color) [40].

**Figure 6:**
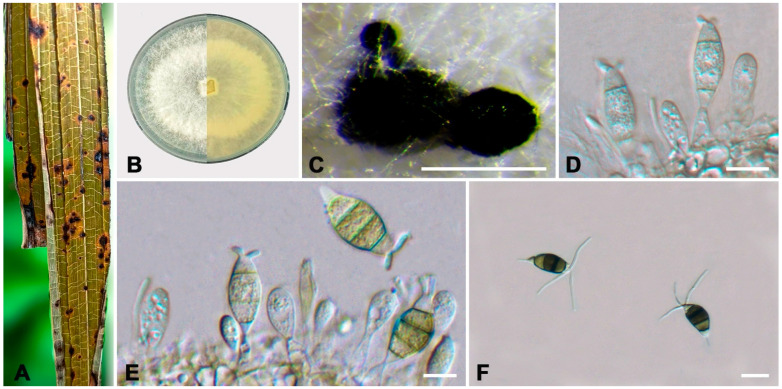
*Neopestalotiopsis concentrica* (living culture CFCC 70629 and CFCC 70619). (**A**) Diseased leaf spots of *Rhapis excelsa*; (**B**) Colony on PDA at seven days; (**C**) Conidial masses formed on PDA; (**D**,**E**) Conidiogenous cells; (**F**) Conidia. Scale bars: (**C**–**F**) = 10 μm.

***Neopestalotiopsis* *concentrica*** C. Peng & C.M. Tian, Persoonia 49: 227, 2022. (Figure 6).

**Description:** See C. Peng et al. [41].

**Material examined:** CHINA, Sichuan Province, Guangyuan City, Chaotian District, 105°55′25″ E, 32°39′11″ N, on leaf spots of *Rhapis excelsa*, Y.X. Li and L. Lin, 10 October 2023 (BJFC-S2341, living culture CFCC 70629). *ibid*. (BJFC-S2342, living culture CFCC 70619).

**Notes:** *N. concentrica* was originally described from spines of *Rosa rugosa* in Henan Province, China [41]. In this study, two isolates (CFCC 70629 and CFCC 70619) clustered together with *N. concentrica* (CFCC 55163, CFCC 55162, and ROC 135) with 67% ML and 0.91% BI value (Figure 2). Therefore, two isolates were identified as *N. concentrica* (Figure 6) as a new host and novel geographic record for China.

**Figure 7:**
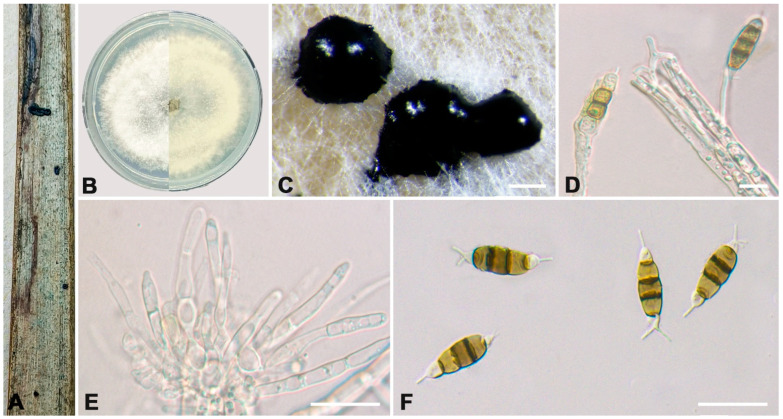
*Neopestalotiopsis phoenicis* (ex-holotype culture CFCC 70625). (**A**) Diseased leaf of *Phoenix canariensis*; (**B**) Colony on PDA at seven days; (**C**) Conidial masses formed on PDA; (**D**,**E**) Conidiogenous cells; (**F**) Conidia. Scale bars: (**C**–**F**) = 10 μm.

***Neopestalotiopsis phoenicis*** W.S. Zhang & X.L. Fan, sp. nov. (Figure 7).

**MycoBank:** MB 854100

**Etymology:** Named after the genus of the host species, *Phoenix*.

**Holotype:** BJFC-S2335

**Description:** Associated with leaf spots of *Phoenix canariensis*. Asexual morph: Conidiomata pycnidial on PDA. Conidiomata are globular, scattered or aggregated, and black. Conidiophores reduce to conidiogenous cells, smooth and hyaline. Conidiogenous cells are discrete, thin-walled, and smooth and hyaline. Conidia are ampulliform, ellipsoid to subcylindrical, straight or slightly curved, smooth, 22.0–28.5 × 5.0–10.0 μm (av. ± SD = 24.36 ± 1.91 × 7.96 ± 1.10 μm, n = 50), L/W = 2.0–4.0 μm (av. ± SD = 3.11 ± 0.45 μm, n = 50), 4-septate; the basal cell is conical to semiellipsoid, with a truncated base, hyaline, smooth, thin-walled, and 3.0–6.0 μm long (av. ± SD = 4.39 ± 0.68 μm, n = 50); three median cells are versicolored, subelliptic to elliptic, with the second cell from the base being honey brown and 4.0–5.5 μm long (av. ± SD = 4.76 ± 0.42 μm, n = 50); the third cell is pale brown or honey brown and 4.0–6.0 μm long (av. ± SD = 4.86 ± 0.53 μm, n = 50); the fourth cell is pale brown and 4.0–6.0 μm long (av. ± SD = 5.04 ± 0.41 μm, n = 50); the apical cell is 1.5–4.0 μm long (av. ± SD = 2.91 ± 0.48 μm, n = 50), hyaline, conic, smooth, and thin-walled, with two or three tubular apical appendages arising from the apical crest, unbranched, filiform, and 3.0–8.0 μm long (av. ± SD = 5.40 ± 1.17 μm, n = 50); a basal appendage is present and 3.0–6.0 μm long (av. ± SD = 4.25 ± 0.72 μm, n = 50). A sexual morph was not observed.

**Culture characteristics:** The colonies on PDA reached up to 55 mm in diameter after seven days at 25 °C. The colonies were filamentous to circular, with dense aerial mycelium on surface, and white from above and reverse. Fruiting bodies were observed after ten days.

**Typus:** CHINA, Sichuan Province, Guangyuan City, Lizhou District, 105°51′23″ E, 32°25′04″ N, on leaf spots of *Phoenix canariensis*, Y.X. Li and X.L. Fan, 9 October 2023 (holotype BJFC-S2335, ex-holotype culture CFCC 70625); *ibid*. (paratype BJFC-S2336, ex-paratype culture CFCC 70621).

**Additional material examined:** CHINA, Sichuan Province, Guangyuan City, Lizhou District, 105°51′23″ E, 32°25′04″ N, on leaf spots of *Phoenix canariensis*, Y.X. Li and X.L. Fan, 9 October 2023 (BJFC-S2337, living culture CFCC 70622).

**Notes:** The three strains of *N. phoenicis* in this study formed a well-supported clade (BI/ML = 0.998/92) (Figure 2). *N. phoenicis* was phylogenetically close to *N. hyperici* (Figure 2), but there were 16 bp different in the concatenated alignment. A comparison of ITS regions showed 10 nucleotide differences with oleaginous *N. hyperici* (509/519, 98.07%). Moreover, *N. phoenicis* could be distinguished from *N. hyperici* by larger conidia (22.0–28.5 vs. 17.0–22.0 μm) and shorter tubular apical appendages (3.0–8.0 vs. 11–23 μm) [20].

**Figure 8:**
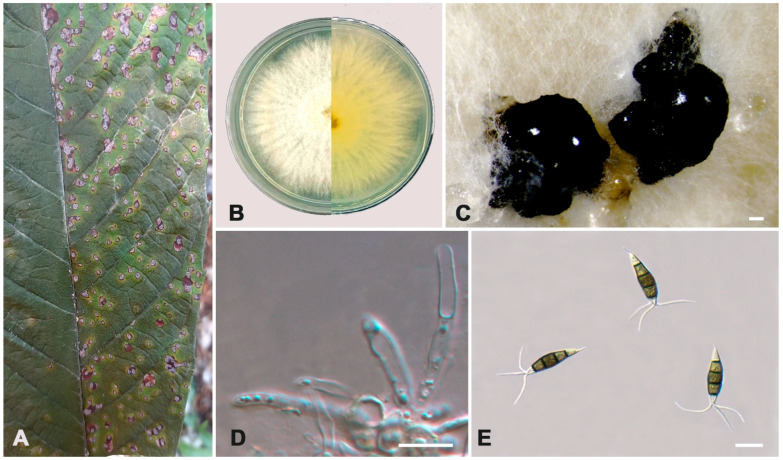
*Pestalotiopsis guiyangensis* (ex-holotype culture CFCC 70626). (**A**) Diseased leaf of *Eriobotrya japonica*; (**B**) Colony on PDA at seven days; (**C**) Conidial masses formed on PDA; (**D**) Conidiogenous cells; (**E**) Conidia. Scale bars: (**C**–**E**) = 10 μm.

***Pestalotiopsis guiyangensis*** W.S. Zhang & X.L. Fan, sp. nov. (Figure 8).

**MycoBank:** MB 854101

**Etymology:** Named for the location of the holotype specimen, Guiyang.

**Holotype:** BJFC-S2343

**Description:** Pathogenic on *Eriobotrya japonica* and *Rohdea japonica* leaves. Asexual morph: Conidiomata pycnidial on PDA. Conidiomata are irregular to globular, scattered or aggregated, and black. Conidiophores reduce to conidiogenous cells, smooth and hyaline. Conidiogenous cells are discrete, thin-walled, and smooth and hyaline. Conidia are fusoid, ellipsoid to subcylindrical, straight or slightly curved, minutely verruculose or smooth, 20.0–27.0 × 4.5–7.0 μm (av. ± SD = 22.9 ± 1.56 × 6.11 ± 0.55 μm, n = 50), L/W = 3.0–5.4 μm (av. ± SD = 3.79 ± 0.47 μm, n = 50), 4-septate; the basal cell is subconical to conical, with a truncated base, hyaline or pale brown, minutely verruculose or smooth, thin-walled, and 3.0–5.5 μm long (av. ± SD = 4.34 ± 0.52 μm, n = 50); three median cells are versicolored, cylindrical, and the second cell from the base is pale brown or brown and 4.0–6.5 μm long (av. ± SD = 5.06 ± 0.44 μm, n = 50); the third cell is honey brown or dark brown and 4.0–5.5 μm long (av. ± SD = 5.06 ± 0.43 μm, n = 50); the fourth cell is pale brown and 4.0–5.0 μm long (av. ± SD = 4.68 ± 0.47 μm, n = 50); the apical cell is 2.5–4.5 μm long (av. ± SD = 3.39 ± 0.38 μm, n = 50), hyaline, conic, smooth, and thin-walled, with two or three tubular apical appendages arising from the apical crest, unbranched, filiform, and 10.5–18.0 μm long (av. ± SD = 15.35 ± 1.76 μm, n = 50); a basal appendage is present, 2.5–6.5 μm long (av. ± SD = 4.81 ± 0.77 μm, n = 50). A sexual morph was not observed.

**Culture characteristics:** The colonies on PDA reached up to 60 mm in diameter after seven days at 25 °C. The colonies were feathery and diffuse, white, with dense aerial mycelium on the surface, and the reverse color was pale yellow. Fruiting bodies were observed after fifteen days.

**Typus:** CHINA, Guizhou Province, Guiyang City, Huaxi District, 106°40′12″ E, 26°25′48″ N, on leaf spots of *Eriobotrya japonica*, Y.X. Li and L. Lin, 18 August 2023 (holotype BJFC-S2343, ex-holotype culture CFCC 70626). CHINA, Guizhou Province, Guiyang City, Huaxi District, 106°40′12″ E, 26°25′48″ N, on leaf spots of *Rohdea japonica*, Y.X. Li and L.L, 18 August 2023 (paratype BJFC-S2344, ex-paratype culture CFCC 70630).

**Notes:** Two *Pestalotiopsis* developed an independent clade in the phylogenetic tree with 85% ML and 0.96 BI value (Figure 3). *P. guiyangensis* was phylogenetically close to *P. oryzae*, but there were nine bp different in the concatenated alignment. The *tef1-α* region showed a five-base difference with oleaginous *P. oryzae* (568/573, 98.12%). In addition, *P. guiyangensis* had a smaller third cell (4.0–5.5 μm) than *P. oryzae* (5.5–7.0 μm), and *P. guiyangensis* could be distinguished from *P. oryzae* by narrower fourth cells (4.0–5.0 vs. 5–6.5 μm) and shorter apical appendages (10.5–18.0 vs. 18–27 μm). In addtion, *P. guiyangensis* had culture characteristics that were different from *P. oryzae* (the *P. guiyangensis* colonies were feathery and diffuse, while *P. oryzae* had an undulate edge, convex with the papilate surface) [15].

## 4. Discussion

In this study, we investigated pestalotioid fungi of plants in Sichuan and Guizhou provinces in China. We included ten sequenced isolates of *Neopestalotiopsis* and two sequenced isolates of *Pestalotiopsis* to provide a backbone tree for the genera *Neopestalotiopsis* and *Pestalotiopsis*. In this study, four new species (*N. acericola*, *N. cercidicola*, *N. phoenicis*, and *P. guiyangensis*) and one known species (*N. concentrica*) were identified on the basis of morphological and phylogenetic analyses.

Pestalotioid fungi are important pathogens of plant diseases, and it has been shown that pestalotioid fungi have no host specificity [42]. For example, *Pestalotiopsis chamaeropis* infests plants in China such as *Quercus acutissima*, *Rosa chinensis*, and *Camellia sinensis* [42]; while *Neopestalotiopsis rhapidis* was found to infest plants in China such as *Rhapis excelsa* and *Podocarpus macrophyllus* [20]. In this study, the coelomycetous fungi were found to infest *Rhapis excelsa* in addition to infesting *Rosa rugosa* in China [41]. The new species, *Pestalotiopsis guiyangensis*, in this study was found to infest different hosts (*Eriobotrya japonica* and *Rohdea japonica*). This indicates that there may be a high undescribed diversity of fungi and host diversity in plants in China [41].

The present study provides a further extension of the backbone tree of *Neopestalotiopsis* and *Pestalotiopsis*, and future work requires further investigation in order to establish a more stable backbone tree of *Neopestalotiopsis* and *Pestalotiopsis*. More sampling is needed in the future to determine the spread and epidemiology of pestalotioid fungi in Guizhou and Sichuan, China. Therefore, future work needs to be carried out on pathogenicity testing and disease control methods, as this can help us to prevent diseases caused by pestalotioid fungi.

Sequence data are essential to resolve these three genera, as many pestalotioid species have overlapping morphological traits [14]. A gene-by-gene assessment of phylogenetic resolution can yield higher levels of protein genes than those of ribosomal regions [43]. Hu et al. [44] suggested that at least two gene combinations (ITS and *tub2*) should be used to resolve the phylogeny of species of *Neopestalotiopsis* and *Pestalotiopsis*. Maharachchikumbura et al. [14] tested ten gene regions to resolve the bound species in *Neopestalotiopsis* and *Pestalotiopsis*, and finally screened the three most applicable regions (ITS, *tef1-α*, and *tub2*) [14]. Liu F et al. [45] investigated a genome-wide phylogenetic tree based on *Colletotrichum*, which could better define the *Colletotrichum* species complex boundaries. Considering the importance of pestalotioid fungi, genome sequencing of pestalotioid species is recommended. This will not only pave the way for comprehensively resolving the tree of life of *Neopestalotiopsis* and *Pestalotiopsis*, but also provide important data for revealing their evolution and adaptive mechanisms and improve our understanding of the genetic basis of various biological features and metabolic potential of these fungi.

## Figures and Tables

**Table 1:** Gene fragments and PCR thermal cycle program used in this study.

Locus	PCR Primers	PCR: Thermal Cycles: (Annealing Temp. in Bold)	Reference
ITS	ITS1/ITS4	(95 °C: 30 s, **51** °C: 30 s, 72 °C: 1 min) × 35 cycles	[33]
*tef1* *-α*	EF1-728F/EF1-1567R	(95 °C: 15 s, **55** °C: 20 s, 72 °C: 1 min) × 35 cycles	[34]
*tub2*	T1/Bt-2b	(95 °C: 30 s, **55** °C: 30 s, 72 °C: 1 min) × 35 cycles	[35]

## Data Availability

The alignments generated during the current study are available in Tree-BASE (accession http://purl.org/phylo/treebase/phylows/study/TB2:S31556, accessed on 7 July 2024). All sequence data are available in NCBI GenBank following the accession numbers in the manuscript.

## Supplementary Materials

The following supporting information can be downloaded at: https://www.mdpi.com/article/10.3390/jof10070475/s1, Table S1: GenBank numbers of all sequences used for phylogenetic analysis in this study.
