# Supplementary material for: Updating the Species Diversity of Pestalotioid Fungi: Four New Species of Neopestalotiopsis and Pestalotiopsis
Source: J Fungi (Basel). 2024 Jul 11;10(7):475. doi: 10.3390/jof10070475 (PMC11278005; doi:10.3390/jof10070475)
Supplement: Supplementary file 1 [file jof-10-00475-s001.zip › Table S1.pdf]

Table S1. GenBank accession numbers of the sequences used for phylogenetic analysis in this study.

Species	Strains	Host	Origin	ITS	*tef1-α*	*tub2*
Neopestalotiopsis acericola	**CFCC 70620**	***Acer palmatum***	**China**	**PP784733**	**PP842622**	**PP842610**
Neopestalotiopsis acericola	**CFCC 70627**	***Acer palmatum***	**China**	**PP784734**	**PP842623**	**PP842611**
Neopestalotiopsis acrostichi	MFLUCC 17-1754 T	*Acrostichum aureum*	Thailand	MK764272	MK764316	MK764338
Neopestalotiopsis acrostichi	MFLUCC 17-1755	*Acrostichum aureum*	Thailand	MK764273	MK764317	MK764339
Neopestalotiopsis alpapicalis	MFLUCC 17-2544 T	*Rhizophora mucronata*	Thailand	MK357772	MK463547	MK463545
Neopestalotiopsis amomi	HKAS 124563	*Amomum villosum*	China	OP498012	OP653489	OP752133
Neopestalotiopsis amomi	HKAS 124564	*Amomum villosum*	China	OP498013	OP753382	OP765913
Neopestalotiopsis aotearoa	CBS 367.54 T	*Canvas*	New Zealand	KM199369	KM199526	KM199454
Neopestalotiopsis asiatica	MFLUCC 12-0286 T	Leaves	China	JX398983	JX399049	JX399018
Neopestalotiopsis australis	CBS 114159 T	*Telopea* sp.	Australia	KM199348	KM199537	KM199432
Neopestalotiopsis brachiata	MFLUCC 17–1555 T	*Rhizophora apiculata*	Thailand	MK764274	MK764318	MK764340
Neopestalotiopsis brasiliensis	COAD 2166 T	*Rhizophora apiculata*	Thailand	MG686469	MG692402	MG692400
Neopestalotiopsis camelliae-oleiferae	CSUFTCC81 T	*Camellia oleifera*	China	OK493585	OK507955	OK562360
Neopestalotiopsis camelliae-oleiferae	CSUFTCC82	*Camellia oleifera*	China	OK493586	OK507956	OK562361
Neopestalotiopsis cavernicola	KUMCC 20-0269 T	Cave rock surface	China	MW545802	MW550735	MW557596
Neopestalotiopsis cavernicola	KUMCC 20-0332	Cave rock surface	China	-	MW590327	MW590328
Neopestalotiopsis cercidicola	**CFCC 70632**	***Cercis canadensis***	**China**	**PP784737**	**PP842626**	**PP842614**
Neopestalotiopsis cercidicola	**CFCC 70624**	***Cercis canadensis***	**China**	**PP784738**	**PP842627**	**PP842615**
Neopestalotiopsis cercidicola	**CFCC 70623**	***Cercis canadensis***	**China**	**PP784739**	**PP842628**	**PP842616**
Neopestalotiopsis chiangmaiensis	MFLUCC 18-0113 T	*Pandanaceae*	Thailand	-	MH388404	MH412725
Neopestalotiopsis chrysea	MFLUCC 12-0261 T	*Pandanus* sp.	China	JX398985	JX399051	JX399020
Neopestalotiopsis chrysea	MFLUCC 12-0262	*Pandanus* sp.	China	JX398986	JX399052	JX399021
Neopestalotiopsis clavispora	MFLUCC 12-0280	*Magnolia* sp.	China	JX398978	JX399044	JX399013
Neopestalotiopsis clavispora	MFLUCC 12-0281 T	*Magnolia* sp.	China	JX398979	JX399045	JX399014
Neopestalotiopsis cocoes	MFLUCC 15-0152 T	*Cocos nucifera*	Thailand	KX789687	KX789689	-
Neopestalotiopsis	HGUP4019 T	*Coffea arabica*	China	KF412649	KF412646	KF412643

coffeae-arabicae						
Neopestalotiopsis concentrica	CFCC 55162	*Rosa rugosa*	China	OK560707	OM622433	OM117698
Neopestalotiopsis concentrica	CFCC 55163	*Rosa chinensis*	China	OK560708	OM622434	OM117699
Neopestalotiopsis concentrica	ROC 135	*Rosa chinensis*	China	OK560709	OM622435	OM117700
Neopestalotiopsis concentrica	**CFCC 70619**	***Rhapis excelsa***	**China**	**PP784735**	**PP842624**	**PP842612**
Neopestalotiopsis concentrica	**CFCC 70629**	***Rhapis excelsa***	**China**	**PP784736**	**PP842625**	**PP842613**
Neopestalotiopsis cubana	CBS 600.96 T	Leaf litter	Cuba	KM199347	KM199521	KM199438
Neopestalotiopsis dendrobii	MFLUCC 14-0106 T	*Dendrobium cariniferum*	Thailand	MK993571	MK975829	MK975835
Neopestalotiopsis drenthii	BRIP 72263a	*Macadamia integrifolia*	Australia	MZ303786	MZ344171	MZ312679
Neopestalotiopsis drenthii	BRIP 72264a T	*Macadamia integrifolia*	Australia	MZ303787	MZ344172	MZ312680
Neopestalotiopsis egyptiaca	CBS 22294 T	*Mangifera indica*	Egypt	KP943747	KP943748	KP943746
Neopestalotiopsis elaeidis	MFLUCC 15-0735 T	*Elaeis guineensis*	Thailand	ON650689	ON734012	-
Neopestalotiopsis elaeagni	HGUP10002	*Elaeagnus pungens*	China	MW930716	MZ203452	MZ683391
Neopestalotiopsis elaeagni	HGUP10004	*Elaeagnus pungens*	China	ON597079	ON595535	ON595537
Neopestalotiopsis ellipsospora	MFLUCC 12-0283 T	Dead plant material	China	JX398980	JX399047	JX399016
Neopestalotiopsis eucalypticola	CBS 264.37 T	*Eucalyptus globulus*	-	KM199376	KM199551	KM199431
Neopestalotiopsis eucalyptorum	MEAN 1308 T	*Eucalyptus globulus*	Portugal	MW794108	MW805397	MW802841
Neopestalotiopsis eucalyptorum	MEAN 1309	*Eucalyptus globulus*	Portugal	MW794098	MW805398	MW802831
Neopestalotiopsis foedans	CGMCC 3.9123 T	Mangrove plant	China	JX398987	JX399053	JX399022
Neopestalotiopsis foedans	CGMCC 3.9178	Mangrove plant	China	JX398989	JX399055	JX399024
Neopestalotiopsis formicarum	CBS 362.72 T	*Photinia serratifolia*	China	KM199358	KM199517	KM199455
Neopestalotiopsis formicarum	GUCC 21-0809	*Photinia serratifolia*	China	OP498007	OP753367	OP752132
Neopestalotiopsis fragariae	ZHKUCC 22-0113 T	*Rosaceae* leaves	China	ON553410	ON569076	ON569075
Neopestalotiopsis guajavae	FMB0026 T	*Psidium guajava*	Pakistan	MF783085	MH460868	MH460871
Neopestalotiopsis guajavae	FMB0027	*Psidium guajava*	Pakistan	MF783084	MH460869	MH460872
Neopestalotiopsis guajavicola	FMB0129 T	*Psidium guajava*	Pakistan	MH209245	MH460870	MH460873
Neopestalotiopsis hadrolaeliae	EHJ7h	*Hadrolaelia jongheana*	Brazil	MK454710	MK465123	MK465121
Neopestalotiopsis haikouensis	SAUCC212271	*Ilex chinensis*	China	OK087294	OK104877	OK104870
Neopestalotiopsis haikouensis	SAUCC212272	*Ilex chinensis*	China	OK087295	OK104878	OK104871
Neopestalotiopsis hispanica	MEAN 1310 T	*Eucalyptus globulus*	Portugal	MW794107	MW805399	MW802840
Neopestalotiopsis honoluluana	CBS 111535	*Telopea* sp.	USA	KM199363	KM199546	KM199461
Neopestalotiopsis honoluluana	CBS 114495 T	*Telopea* sp.	USA	KM199364	KM199548	KM199457

Neopestalotiopsis hydeana	MFLUCC 20–0132 T	*Artocarpus heterophyllus*	Thailand	MW266069	MW251129	MW251119
Neopestalotiopsis hyperici	KUNCC 22-12597	*Hypericum monogynum*	China	OP498010	OP713768	OP765908
Neopestalotiopsis hyperici	KUNCC 22-12598	*Hypericum monogynum*	China	OP498009	OP737880	OP737883
Neopestalotiopsis iberica	MEAN 1313 T	*Eucalyptus globulus*	Portugal	MW794111	MW805402	MW802844
Neopestalotiopsis iberica	MEAN 1314	*Eucalyptus globulus*	Portugal	MW794114	MW805403	MW802847
Neopestalotiopsis iranensis	CBS 137768 T	*Fragaria* x *ananassa*	Iran	KM074048	KM074051	KM074057
Neopestalotiopsis javaensis	CBS 257.31 T	*Cocos nucifera*	Indonesia	KM199357	KM199543	KM199437
Neopestalotiopsis longiappendiculata	MEAN 1315 T	*Eucalyptus globulus*	Portugal	MW794112	MW805404	MW802845
Neopestalotiopsis longiappendiculata	MEAN 1316	*Eucalyptus globulus*	Portugal	MW794103	MW805405	MW802836
Neopestalotiopsis lusitanica	MEAN 1317 T	*Eucalyptus globulus*	Portugal	MW794110	MW805406	MW802843
Neopestalotiopsis macadamiae	BRIP 63737c T	*Macadamia integrifolia*	New South Wales	KX186604	KX186627	KX186654
Neopestalotiopsis maddoxii	BRIP 72262a T	*Macadamia integrifolia*	Australia	MZ303781	MZ344166	MZ312674
Neopestalotiopsis maddoxii	BRIP 72266a	*Macadamia integrifolia*	Australia	MZ303782	MZ344167	MZ312675
Neopestalotiopsis magna	MFLUCC 12-652 T	*Pteridium* sp.	France	KF582795	KF582791	KF582793
Neopestalotiopsis mesopotamica	CBS 336.86 T	*Pinus brutia*	Iraq	KM199362	KM199555	KM199441
Neopestalotiopsis mianyangensis	CGMCC 3.23555 T	*Paeonia suffruticosa*	China	OP546681	OP723490	OP672161
Neopestalotiopsis mianyangensis	UESTCC 22.0006	*Paeonia suffruticosa*	China	OP082291	OP204793	OP235979
Neopestalotiopsis musae	MFLUCC 15-0776 T	*Musa* sp.	Thailand	KX789683	KX789685	KX789686
Neopestalotiopsis natalensis	CBS 138.41 T	*Acacia mollissima*	South Africa	KM199377	KM199552	KM199466
Neopestalotiopsis nebuloides	BRIP 66617 T	*Sporobolus elongatus*	Australia	MK966338	MK977633	MK977632
Neopestalotiopsis olumideae	BRIP 72273a T	*Macadamia integrifolia*	Australia	MZ303790	MZ344175	MZ312683
Neopestalotiopsis olumideae	BRIP 72283a	*Macadamia integrifolia*	Australia	MZ303791	MZ344176	MZ312684
Neopestalotiopsis paeoniae-suffruticosae	CGMCC 3.23554 T	*Paeonia suffruticosa*	China	OP082292	OP204794	OP235980
Neopestalotiopsis paeoniae-suffruticosae	UESTCC 22.0007	*Paeonia suffruticosa*	China	OP082293	OP204795	OP235981
Neopestalotiopsis pandanicola	MFLUCC 22-0144	*Pandanus* sp.	–	OP802391	OP830890	OP801768
Neopestalotiopsis pernambucana	RV02	*Vismia guianensis*	Brazil	KJ792467	KU306740	-
Neopestalotiopsis pernambucana	URM7148 T	*Vismia guianensis*	Brazil	KJ792466	KU306739	–
Neopestalotiopsis perukae	FMB0127 T	*Psidium guajava*	Pakistan	MH209077	MH523647	MH460876

Neopestalotiopsis perukae	FMB0128	*Psidium guajava*	Pakistan	MH209246	MH523646	MH460875
Neopestalotiopsis petila	MFLUCC 17-1737 T	*Rhizophora mucronata*	Thailand	MK764275	MK764319	MK764341
Neopestalotiopsis petila	MFLUCC 17-1738	*Rhizophora mucronata*	Thailand	MK764276	MK764320	MK764342
Neopestalotiopsi. phangngaensis	MFLUCC 18-0119	*Pandanus* sp.	–	MH388354	MH388390	MH412721
Neopestalotiopsis phoenicis	**CFCC 70625**	***Phoenix canariensis***	**China**	**PP784730**	**PP842619**	**PP842607**
Neopestalotiopsis phoenicis	**CFCC 70621**	***Phoenix canariensis***	**China**	**PP784731**	**PP842620**	**PP842608**
Neopestalotiopsis phoenicis	**CFCC 70622**	***Phoenix canariensis***	**China**	**PP784732**	**PP842621**	**PP842609**
Neopestalotiopsis photiniae	MFLUCC 22-0129	*Photinia serrulate*	China	OP498008	OP753368	OP752131
Neopestalotiopsis photiniae	GUCC 21-0820	*Photinia serrulate*	China	OP806524	OP828691	OP896200
Neopestalotiopsis piceana	CBS 254.32	*Picea* sp.	UK	KM199372	KM199529	KM199452
Neopestalotiopsis piceana	CBS 394.48 T	*Picea* sp.	UK	KM199368	KM199527	KM199453
Neopestalotiopsis protearum	CBS 114178 T	*Leucospermum cuneiforme*	Zimbabwe	JN712498	LT853201	KM199463
Neopestalotiopsis psidii	FMB0028 T	*Psidium guajava*	Pakistan	MF783082	MH460874	MH477870
Neopestalotiopsis rhapidis	KUNCC 22-12590	*Podocarpus macrophyllus*	China	OP498004	OP753369	OP752134
Neopestalotiopsis rhizophorae	MFLUCC 17-1550 T	*Rhizophora mucronata*	Thailand	MK764278	MK764322	MK764344
Neopestalotiopsis rhododendri	GUCC 21504 T	*Rhododendron simsii*	China	MW979577	MW980444	MW980443
Neopestalotiopsis rhododendricola	KUN-HKAS 123204	*Rhododendron* sp.	China	OK283069	OK274148	OK274147
Neopestalotiopsis rosae	CBS 101057 T	*Rosa* sp.	New Zealand	KM199359	KM199523	KM199429
Neopestalotiopsis rosae	CBS 124745	*Paeonia suffruticosa*	USA	KM199360	KM199524	KM199430
Neopestalotiopsis rosicola	CFCC 51992 T	*Rosa chinensis*	China	KY885239	KY885243	KY885245
Neopestalotiopsis rosicola	CFCC 51993	*Rosa chinensis*	China	KY885240	KY885244	KY885246
Neopestalotiopsis samarangensis	CBS 115451 T	*Syzygium samarangense*	Thailand	KM199365	KM199556	KM199447
Neopestalotiopsis saprophytica	MFLUCC 12-0282 T	*Magnolia* sp.	China	JX398982	JX399048	JX399017
Neopestalotiopsis scalabiensis	CAA1029	*Vaccinium corymbosum*	Portugal	MW969748	MW959100	MW934611
Neopestalotiopsis sichuanensis	CFCC 54338 T	*Castanea mollissima*	China	MW166231	MW199750	MW218524
Neopestalotiopsis sichuanensis	SM15-1C	*Castanea mollissima*	China	MW166232	MW199751	MW218525
Neopestalotiopsis siciliana	AC46	*Persea americana*	Italy	ON117813	ON107273	ON209162
Neopestalotiopsis siciliana	AC48	*Persea americana*	Italy	ON117812	ON107274	ON209163

Neopestalotiopsis siciliana	AC49	*Persea americana*	Italy	ON117811	ON107275	ON209164
Neopestalotiopsis sonneratae	MFLUCC 17-1744	*Sonneronata alba*	Thailand	MK764279	MK764323	MK764345
Neopestalotiopsis sonneratae	MFLUCC 17-1745 T	*Sonneronata alba*	Thailand	MK764280	MK764324	MK764346
Neopestalotiopsis subepidermalis	CFCC 55160 T	*Rosa rugosa*	China	OK560699	OM622425	OM117690
Neopestalotiopsis subepidermalis	CFCC 55161	*Rosa chinensis*	China	OK560701	OM622427	OM117692
Neopestalotiopsis subepidermalis	ROC 170	*Rosa chinensis*	China	OK560702	OM622428	OM117693
Neopestalotiopsis suphanburiensis	MFLUCC 22-0126	unidentified plant	Thailand	OP497994	OP753372	OP752135
Neopestalotiopsis sp.	CSUFTCC61	*Camellia oleifera*	China	OK493590	OK507960	OK562365
Neopestalotiopsis sp.	CSUFTCC62	*Camellia oleifera*	China	OK493591	OK507961	OK562366
Neopestalotiopsis sp.	CSUFTCC63	*Camellia oleifera*	China	OK493592	OK507962	OK562367
Neopestalotiopsis sp. 1	CFCC 54337	*Camellia oleifera*	China	MW166233	MW199752	MW218526
Neopestalotiopsis sp. 1	ZX12-1	*Camellia oleifera*	China	MW166234	MW199753	MW218527
Neopestalotiopsis steyaertii	IMI 192475 T	*Eucalyptus viminalis*	Australia	KF582796	KF582792	KF582794
Neopestalotiopsis surinamensis	CBS 450.74 T	Soil	Suriname	KM199351	KM199518	KM199465
Neopestalotiopsis terricola	CGMCC 3.23553 T	*Paeonia suffruticosa*	China	OP082294	OP204796	OP235982
Neopestalotiopsis thailandica	MFLUCC 17-1730 T	*Rhizophora mucronata*	Thailand	MK764281	MK764325	MK764347
Neopestalotiopsis thailandica	MFLUCC 17-1731	*Rhizophora mucronata*	Thailand	MK764282	MK764326	MK764348
Neopestalotiopsis umbrinospora	MFLUCC 12-0285 T	Dead leaves	China	JX398984	JX399050	JX399019
Neopestalotiopsis vaccinii	CAA1059 T	*Vaccinium corymbosum*	Portugal	MW969747	MW959099	MW934610
Neopestalotiopsis vacciniicola	CAA1053	*Vaccinium corymbosum*	Portugal	MW969749	MW959101	MW934612
Neopestalotiopsis vacciniicola	CAA1054	*Vaccinium corymbosum*	Portugal	MW969750	MW959102	MW934613
Neopestalotiopsis vheenae	BRIP 72293a T	*Macadamia integrifolia*	Australia	MZ303792	MZ344177	MZ312685
Neopestalotiopsis vitis	MFLUCC 15-1265 T	*Vitis vinifera*	China	KU140694	KU140676	KU140685
Neopestalotiopsis vitis	MFLUCC 15-1270	*Vitis vinifera*	China	KU140699	KU140681	KU140690
Neopestalotiopsis xishuangbannaensis	KUMCC 21-0424 T	*Kerivoula hardwickii*	China	ON426865	OR025973	OR025934
Neopestalotiopsis xishuangbannaensis	KUMCC 21-0425	*Kerivoula hardwickii*	China	ON426866	OR025974	OR025935
Neopestalotiopsis zakeelii	BRIP 72271a	*Macadamia integrifolia*	Australia	MZ303788	MZ344173	MZ312681
Neopestalotiopsis zakeelii	BRIP 72282a T	*Macadamia integrifolia*	Australia	MZ303789	MZ344174	MZ312682
Neopestalotiopsis zimbabwana	CBS 111495 T	*Leucospermum cunciforme*	Zimbabwe	JX556231	KM199545	KM199456

Neopestalotiopsis zingiberis	HGUP10001	*Zingiber officinale*	China	MW930715	MZ683389	MZ683390
Neopestalotiopsiszingiberis	HGUP10005	*Zingiber officinale*	China	ON597078	ON595536	ON595538
Pestalotiopsis abietis	CFCC 53011 T	*Abies fargesii*	China	MK397013	MK622277	MK622280
Pestalotiopsis abietis	CFCC 53012	*Abies fargesii*	China	MK397014	MK622278	MK622281
Pestalotiopsis adusta	ICMP 6088 T	refrigerator door	Fiji	JX399006	JX399070	JX399037
Pestalotiopsis adusta	MFLUCC 10-146	*Syzygium* sp.	Thailand	JX399007	JX399071	JX399038
Pestalotiopsis aggestorum	LC6301 T	*Camellia sinensis*	China	KX895015	KX895234	KX895348
Pestalotiopsis aggestorum	LC8186	*Camellia sinensis*	China	KY464140	KY464150	KY464160
Pestalotiopsis anacardiacearum	IFRDCC 2397 T	*Mangifera indica*	China	KC247154	KC247156	KC247155
Pestalotiopsis anhuiensis	CFCC 54791 T	*Cyclobalanopsis glauca*	China	ON007028	ON005045	ON005056
Pestalotiopsis arceuthobii	CBS 434.65 T	*Arceuthobium campylopodum*	USA	KM199341	KM199516	KM199427
Pestalotiopsis arenga	CBS 331.92 T	*Arenga undulatifolia*	Singapore	KM199340	KM199515	KM199426
Pestalotiopsis australasiae	CBS 114126 T	*Knightia* sp.	New Zealand	KM199297	KM199499	KM199409
Pestalotiopsis australasiae	CBS 114141	*Protea* sp.	New South Wales	KM199298	KM199501	KM199410
Pestalotiopsis australis	CBS 111503	*Protea neriifolia × susannae*	South Africa	KM199331	KM199557	KM199382
Pestalotiopsis australis	CBS 114193 T	*Grevillea sp.*	New South Wales	KM199332	KM199475	KM199383
Pestalotiopsis biciliata	CBS 124463 T	*Platanus × hispanica*	Slovakia	KM199308	KM199505	KM199399
Pestalotiopsis biciliata	CBS 236.38	*Paeonia* sp.	Italy	KM199309	KM199506	KM199401
Pestalotiopsis brachiata	LC2988 T	*Camellia* sp.	China	KX894933	KX895150	KX895265
Pestalotiopsis brachiata	LC8188	*Camellia* sp.	China	KY464142	KY464152	KY464162
Pestalotiopsis brachiata	LC8189	*Camellia* sp.	China	KY464143	KY464153	KY464163
Pestalotiopsis brassicae	CBS 170.26 T	*Brassica napus*	New Zealand	KM199379	KM199558	–
Pestalotiopsis camelliae	MFLUCC 12-0277 T	*Camellia japonica*	China	JX399010	JX399074	JX399041
Pestalotiopsis camelliae-japonicae	ZHKUCC23-0826	*Camellia japonica*	China	OR258040	OR251480	OR251483
Pestalotiopsis camelliae-oleiferae	CSUFTCC08 T	*Camelliae oleiferae*	China	OK493593	OK507963	OK562368
Pestalotiopsis camelliae-oleiferae	CSUFTCC09	*Camelliae oleiferae*	China	OK493594	OK507964	OK562369
Pestalotiopsis cangshanensis	CGMCC 3.23544	*Rhododendron delavayi*	China	OP082426	OP185510	OP185517
Pestalotiopsis castanopsidis	CFCC 54430 T	*Castanopsis lamontii*	China	OK339732	OK358493	OK358508
Pestalotiopsis castanopsidis	CFCC 54305	*Castanopsis hystrix*	China	OK339733	OK358494	OK358509

Pestalotiopsis castanopsidis	CFCC 54384	*Castanopsis hystrix*	China	OK339734	OK358495	OK358510
Pestalotiopsis chamaeropis	CBS 186.71 T	*Chamaerops humilis*	Italy	KM199326	KM199473	KM199391
Pestalotiopsis chamaeropis	LC3619	*Camellia* sp.	China	KX894991	KX895208	KX895322
Pestalotiopsis chamaeropis	CFCC 54776	*Quercus variabilis*	China	OM746234	OM840006	OM839907
Pestalotiopsis chamaeropis	CFCC 55338	*Quercus variabilis*	China	OM746235	OM840007	OM839908
Pestalotiopsis changjiangensis	CFCC 54314 T	*Castanopsis tonkinensis*	China	OK339739	OK358500	OK358515
Pestalotiopsis changjiangensis	CFCC 54433	*Castanopsis hainanensis*	China	OK339740	OK358501	OK358516
Pestalotiopsis changjiangensis	CFCC 52803	*Cyclobalanopsis austrocochinchinensis*	China	OK339741	OK358502	OK358517
Pestalotiopsis chaoyangensis	CFCC55549 T	*Euonymus japonicus*	China	OQ344763	OQ410582	OQ410584
Pestalotiopsis chaoyangensis	CFCC58805	*Euonymus japonicus*	China	OQ344764	OQ410583	OQ410585
Pestalotiopsis chiangmaiensis	MFLUCC 22-0127	Bamboo	Thailand	OP497990	OP753374	OP752137
Pestalotiopsis chiaroscuro	BRIP 72970	*Sporobolus natalensis*	Australia	OK422510	OK423753	OK423752
Pestalotiopsis clavata	MFLUCC 12-0268 T	*Buxus* sp.	China	JX398990	JX399056	JX399025
Pestalotiopsis colombiensis	CBS 118553 T	*Eucalyptus eurograndis*	Colombia	KM199307	KM199488	KM199421
Pestalotiopsis cyclobalanopsidis	CFCC 54328 T	*Cyclobalanopsis glauca*	China	OK339735	OK358496	OK358511
Pestalotiopsis cyclobalanopsidis	CFCC 55891	*Cyclobalanopsis glauca*	China	OK339736	OK358497	OK358512
Pestalotiopsis digitalis	MFLU 14-0208 T	*Digitalis purpurea*	New Zealand	KP781879	–	KP781883
Pestalotiopsis dilucida	LC3232	*Camellia sinensis*	China	KX894961	KX895178	KX895293
Pestalotiopsis dilucida	LC8184	*Camellia sinensis*	China	KY464138	KY464148	KY464158
Pestalotiopsis diploclisiae	CBS 115449	*Psychotria tutcheri*	China	KM199314	KM199485	KM199416
Pestalotiopsis diploclisiae	CBS 115587 T	*Diploclisia glaucescens*	China	KM199320	KM199486	KM199419
Pestalotiopsis disseminata	CBS 143904	*Persea americana*	New Zealand	MH554152	MH554587	MH554825
Pestalotiopsis disseminata	MEAN 1165	*Pinus pinea*	Portugal	MT374687	MT374699	MT374712
Pestalotiopsis diversiseta	MFLUCC 12-0287 T	*Rhododendron* sp.	China	JX399009	JX399073	JX399040
Pestalotiopsis doitungensis	MFLUCC 14-0115 T	*Dendrobium* sp.	Thailand	MK993574	MK975832	MK975837
Pestalotiopsis dracaenicola	MFLUCC 18-0913 T	*Dracaena* sp.	Thailand	MN962731	MN962732	MN962733
Pestalotiopsis dracontomelon	MFLU 14-0207 T	*Dracontomelon dao*	Thailand	KP781877	KP781880	–
Pestalotiopsis eleutherococci	HMJAU 60190	*Eleutherococcus brachypus*	China	OL996127	-	OL898722
Pestalotiopsis endophytica	MFLUCC 18-0932	*Magnolia garrettii*	Thailand	NR_172439	MW417119	-
Pestalotiopsis ericacearum	IFRDCC 2439 T	*Rhododendron delavayi*	China	KC537807	KC537814	KC537821
Pestalotiopsis etonensis	BRIP 66615 T	*Sporobolus jacquemontii*	Australia	MK966339	MK977635	MK977634

Pestalotiopsis ficicola	SAUCC230046 T	*Ficus microcarpa*	China	OQ691974	OQ718691	OQ718749
Pestalotiopsis ficicola	SAUCC230042	*Ficus microcarpa*	China	OQ691972	OQ718689	OQ718747
Pestalotiopsis ficicola	SAUCC230043	*Ficus microcarpa*	China	OQ691973	OQ718690	OQ718748
Pestalotiopsis ficicrescens	GUCC 21556	*Camellia japonica*	China	MZ477311	-	MZ868301
Pestalotiopsis foliicola	CFCC 54440 T	*Castanopsis faberi*	China	ON007029	ON005046	ON005057
Pestalotiopsis foliicola	CFCC 57359	*Castanopsis faberi*	China	ON007030	ON005047	ON005058
Pestalotiopsis foliicola	CFCC 57360	*Castanopsis faberi*	China	ON007031	ON005048	ON005059
Pestalotiopsis formosana	NTUCC 17-009 T	*Poaceae* sp.	China	MH809381	MH809389	MH809385
Pestalotiopsis furcata	MFLUCC 12-0054 T	*Camellia sinensis*	Thailand	JQ683724	JQ683740	JQ683708
Pestalotiopsis furcata	LC6691	*Camellia sinensis*	China	KX895030	KX895248	KX895363
Pestalotiopsis fusoidea	CGMCC 3.23545	*Rhododendron delavayi*	China	OP082427	OP185512	OP185519
Pestalotiopsis gaultheriae	IFRD 411-014 T	*Gaultheria forrestii*	China	KC537805	KC537812	KC537819
Pestalotiopsis gibbosa	NOF 3175 T	*Gaultheria shallon*	Canada	LC311589	LC311591	LC311590
Pestalotiopsis grevilleae	CBS 114127 T	*Grevillea* sp.	Australia	KM199300	KM199504	KM199407
Pestalotiopsis guangdongensis	ZHKUCC 22 0016 T	*Arenga pinnata*	China	ON180762	ON221520	ON221548
Pestalotiopsis guangxiensis	CFCC 54308 T	*Quercus griffithii*	China	OK339737	OK358498	OK358513
Pestalotiopsis guangxiensis	CFCC 54300	*Quercus griffithii*	China	OK339738	OK358499	OK358514
Pestalotiopsis guiyangensis	**CFCC 70626**	***Eriobotrya japonicac***	**China**	**PP784740**	**PP842629**	**PP842617**
Pestalotiopsis guiyangensis	**CFCC 70630**	***Rohdea japonica***	**China**	**PP784741**	**PP842630**	**PP842618**
Pestalotiopsis guizhouensis	CFCC 54803 T	*Cyclobalanopsis glauca*	China	ON007035	ON005052	ON005063
Pestalotiopsis guizhouensis	CFCC 57364	*Cyclobalanopsis glauca*	China	ON007036	ON005053	ON005064
Pestalotiopsis hawaiiensis	CBS 114491 T	*Leucospermum* sp.	USA	KM199339	KM199514	KM199428
Pestalotiopsis hispanica	CBS 115391 T	*Protea* sp.	Spain	MH553981	MH554399	MH554640
Pestalotiopsis hispanica	KUNCC 22-12595	*Protea* sp.	Spain	OP498001	OP753381	OP765910
Pestalotiopsis hispanica	KUNCC 22-12593	*Protea* sp.	Spain	OP498000	OP753378	OP737882
Pestalotiopsis hispanica	KUNCC 22-12594	*Protea* sp.	Spain	OP498002	OP753380	OP765912
Pestalotiopsis hollandica	CBS 265.33 T	*Sciadopitys verticillata*	Netherlands	KM199328	KM199481	KM199388
Pestalotiopsis humicola	CBS 336.97 T	soil	Papua New Guinea	KM199317	KM199484	KM199420
Pestalotiopsis hunanensis	CSUFTCC15 T	*Camellia oleifera*	China	OK493599	OK507969	OK562374
Pestalotiopsis hunanensis	CSUFTCC18	*Camellia oleifera*	China	OK493600	OK507970	OK562375
Pestalotiopsis hydei	MFLUCC 20-0135	*Litsea petiolate*	China	NR_172003	MW251113	MW251112

Pestalotiopsis hydei	GUCC 21-0816	*Litsea petiolate*	China	OP753660	OP753383	OP765909
Pestalotiopsis iberica	CAA1006	*Pinus radiata*	Spain	MW732249	MW759039	MW759036
Pestalotiopsis inflexa	MFLUCC 12-0270 T	Unidentified tree	China	JX399008	JX399072	JX399039
Pestalotiopsis intermedia	MFLUCC 12-0259 T	Unidentified tree	China	JX398993	JX399059	JX399028
Pestalotiopsis italiana	MFLU 14-0214 T	*Cupressus glabra*	Italy	KP781878	KP781881	KP781882
Pestalotiopsis jiangsuensis	CFCC 59539	*Pinus massoniana*	China	OR533578	OR539187	OR539192
Pestalotiopsis jiangsuensis	CFCC 59540	*Pinus massoniana*	China	OR533579	OR539188	OR539193
Pestalotiopsis jiangxiensis	LC4399 T	*Camellia* sp.	China	KX895009	KX895227	KX895341
Pestalotiopsis jinchanghensis	LC6636 T	*Camellia sinensis*	China	KX895028	KX895247	KX895361
Pestalotiopsis jinchanghensis	LC8190	*Camellia sinensis*	China	KY464144	KY464154	KY464164
Pestalotiopsis kaki	KNU-PT-1804 T	*Diospyros kaki*	Korea	LC552953	LC553555	LC552954
Pestalotiopsis kandelicola	NCYU 19-0355 T	*Kandelia candel*	China	MT560723	MT563102	MT563100
Pestalotiopsis kenyana	CBS 442.67 T	*Coffea* sp.	Kenya	KM199302	KM199502	KM199395
Pestalotiopsis knightiae	CBS 111963	*Knightia* sp.	New Zealand	KM199311	KM199495	KM199406
Pestalotiopsis knightiae	CBS 114138 T	*Knightia* sp.	New Zealand	KM199310	KM199497	KM199408
Pestalotiopsis krabiensis	MFLUCC 16-0260 T	*Pandanus* sp.	Thailand	MH388360	MH388395	MH412722
Pestalotiopsis leucadendri	CBS 121417 T	*Leucadendron* sp.	South Africa	MH553987	MH554412	MH554654
Pestalotiopsis licualicola	HGUP 4057 T	*Licuala grandis*	China	KC492509	KC481684	KC481683
Pestalotiopsis linearis	MFLUCC 12-0271 T	*Trachelospermum* sp.	China	JX398992	JX399058	JX399027
Pestalotiopsis linguae	ZHKUCC22-0159	*Pyrrosia lingua*	China	OP094104	OP186110	OP186108
Pestalotiopsis lithocarpi	CFCC 55100 T	*Lithocarpus chiungchungensis*	China	OK339742	OK358503	OK358518
Pestalotiopsis lithocarpi	CFCC 55893	*Lithocarpus chiungchungensis*	China	OK339743	OK358504	OK358519
Pestalotiopsis loeiana	MFLUCC 22-0123	Unkonwed	Thailand	OP497988	OP737881	OP713769
Pestalotiopsis lushanensis	LC4344 T	*Camellia* sp.	China	KX895005	KX895223	KX895337
Pestalotiopsis lushanensis	LC8182	*Camellia* sp.	China	KY464136	KY464146	KY464156
Pestalotiopsis lushanensis	LC8183	*Camellia* sp.	China	KY464137	KY464147	KY464157
Pestalotiopsis lushanensis	CFCC 54894	*Quercus serrata*	China	OM746282	OM840054	OM839955
Pestalotiopsis macadamiae	BRIP 63738b	*Macadamia integrifolia*	Australia	KX186588	KX186621	KX186680
Pestalotiopsis macadamiae	BRIP 63739b	*Macadamia integrifolia*	Australia	KX186587	KX186620	KX186679
Pestalotiopsis malayana	CBS 102220 T	*Macaranga triloba*	Malaysia	KM199306	KM199482	KM199411
Pestalotiopsis manyueyuanani	NTUPPMCC 18-165	*Ophiocordyceps* sp. *parasitic*	China	OR125060	OR126313	OR126306
Pestalotiopsis manyueyuanani	NTUPPMCC 22-012	*Ophiocordyceps* sp. *parasitic*	China	OR125061	OR126314	OR126307

Pestalotiopsis monochaeta	CBS 144.97 T	*Quercus robur*	Netherlands	KM199327	KM199479	KM199386
Pestalotiopsis monochaeta	CBS 440.83	*Taxus baccata*	Netherlands	KM199329	KM199480	KM199387
Pestalotiopsis montellica	MFLUCC 12-0279	*Fagraea bodeni*	China	JX399012	JX399076	JX399043
Pestalotiopsis multicolor	CFCC59981	*Taxus chinensis*	China	OQ626676	OQ714341	OQ714336
Pestalotiopsis nanjingensis	CFCC 53882	*Quercus aliena*	China	OM746295	OM840067	OM839968
Pestalotiopsis nanjingensis	CSUFTCC16 T	*Camellia oleifera*	China	OK493602	OK507972	OK562377
Pestalotiopsis nanjingensis	CSUFTCC20	*Camellia oleifera*	China	OK493603	OK507973	OK562378
Pestalotiopsis nanningensis	CSUFTCC10 T	*Camellia oleifera*	China	OK493596	OK507966	OK562371
Pestalotiopsis nanningensis	CSUFTCC11	*Camellia oleifera*	China	OK493597	OK507967	OK562372
Pestalotiopsis neolitseae	NTUCC 17-011 T	*Neolitsea villosa*	China	MH809383	MH809391	MH809387
Pestalotiopsis neolitseae	CFCC 54590	*Lithocarpus amygdalifolius*	China	OK339744	OK358505	OK358520
Pestalotiopsis novae-hollandiae	CBS 130973 T	*Banksia grandis*	Australia	KM199337	KM199511	KM199425
Pestalotiopsis oryzae	CBS 111522	*Telopea* sp.	USA	KM199294	KM199493	KM199394
Pestalotiopsis oryzae	CBS 171.26	NA	Italy	KM199304	KM199494	KM199397
Pestalotiopsis oryzae	CBS 353.69 T	*Oryza sativa*	Denmark	KM199299	KM199496	KM199398
Pestalotiopsis pallidotheae	MAFF 240993 T	*Pieris japonica*	Japan	AB482220	–	–
Pestalotiopsis pandanicola	MFLUCC 16-0255 T	*Pandanus* sp.	Thailand	MH388361	MH388396	MH412723
Pestalotiopsis papuana	CBS 331.96 T	coastal soil	Papua New Guinea	KM199321	KM199491	KM199413
Pestalotiopsis papuana	CBS 887.96	*Cocos nucifera*	Papua New Guinea	KM199318	KM199492	KM199415
Pestalotiopsis parva	CBS 265.37	*Delonix regia*	NA	KM199312	KM199508	KM199404
Pestalotiopsis parva	CBS 278.35 T	*Delonix regia*	NA	KM199313	KM199509	KM199405
Pestalotiopsis phoebes	SAUCC230093 T	*Phoebe zhennan*	China	OQ692028	OQ718745	OQ718803
Pestalotiopsis photiniicola	GZCC 16-0028*	*Photinia serrulata*	China	KY092404	KY047662	KY047663
Pestalotiopsis pini	MEAN 1092	*Pinus pinea*	Portugal	MT374680	MT374693	MT374705
Pestalotiopsis pinicola	KUMCC 19-0183 T	*Pinus armandii*	China	MN412636	MN417509	MN417507
Pestalotiopsis portugalica	CBS 393.48 T	NA	Portugal	KM199335	KM199510	KM199422
Pestalotiopsis pyrrosiae-linguae	ZHKUCC23-0807	*Pyrrosia lingua*	China	OR199902	OR259260	OR259258
Pestalotiopsis rhizophorae	MFLUCC 17-0416 T	*Rhizophora mucronata*	Thailand	MK764283	MK764327	MK764349
Pestalotiopsis rhizophorae	MFLUCC 17-0417	*Rhizophora mucronata*	Thailand	MK764284	MK764328	MK764350
Pestalotiopsis rhododendri	IFRDCC 2399 T	*Rhododendron sinogrande*	China	KC537804	KC537811	KC537818

Pestalotiopsis rhodomyrtus	CFCC 54733	*Quercus aliena*	China	OM746310	OM840082	OM839983
Pestalotiopsis rhodomyrtus	CFCC 55052	*Cyclobalanopsis augustinii*	China	OM746311	OM840083	OM839984
Pestalotiopsis rosarioides	CGMCC 3.23549	*Rhododendron decorum*	China	OP082430	OP185513	OP185520
Pestalotiopsis rosea	MFLUCC 12-0258 T	*Pinus* sp.	China	JX399005	JX399069	JX399036
Pestalotiopsis Sabal	ZHKUCC 22－0035 T	*Sabal mexicana*	China	ON180775	ON221533	ON221561
Pestalotiopsis scoparia	CBS 176.25 T	*Chamaecyparis* sp.	China	KM199330	KM199478	KM199393
Pestalotiopsis sequoiae	MFLUCC 13-0399 T	*Sequoia sempervirens*	Italy	KX572339	–	–
Pestalotiopsis shaanxiensis	CFCC 54958 T	*Quercus variabilis*	China	ON007026	ON005043	ON005054
Pestalotiopsis shaanxiensis	CFCC 57356	*Quercus variabilis*	China	ON007027	ON005044	ON005055
Pestalotiopsis shorea	MFLUCC 12-0314	*Shorea obtusa*	Thailand	KJ503811	KJ503817	KJ503814
Pestalotiopsis silvicola	CFCC 55296 T	*Cyclobalanopsis kerrii*	China	ON007032	ON005049	ON005060
Pestalotiopsis silvicola	CFCC 54915	*Cyclobalanopsis kerrii*	China	ON007033	ON005050	ON005061
Pestalotiopsis silvicola	CFCC 57363	*Cyclobalanopsis kerrii*	China	ON007034	ON005051	ON005062
Pestalotiopsis smilacicola	MFLUCC 22-0124	*Smilax china, Dioscorea* sp.	Thailand	OP497989	OP737879	OP762674
Pestalotiopsis smilacicola	MFLUCC 22-0125	*Smilax china, Dioscorea* sp.	Thailand	OP497991	OP753376	OP762673
Pestalotiopsis sonneratiae	CFCC 57394	*Sonneratia*	China	ON114184	ON086812	ON086816
Pestalotiopsis sonneratiae	CFCC 57395	*Sonneratia*	China	ON114185	ON086813	ON086817
Pestalotiopsis spatholobi	SAUCC231201 T	*Spatholobus suberectus*	China	OQ692023	OQ718740	OQ718798
Pestalotiopsis spatholobi	SAUCC231203	*Spatholobus suberectus*	China	OQ692024	OQ718741	OQ718799
Pestalotiopsis spatholobi	SAUCC231204	*Spatholobus suberectus*	China	OQ692025	OQ718742	OQ718800
Pestalotiopsis spathulata	CBS 356.86 T	*Gevuina avellana*	Chile	KM199338	KM199513	KM199423
Pestalotiopsis spathuliappendiculata	CBS 144035 T	*Phoenix canariensis*	Australia	MH554172	MH554607	MH554845
Pestalotiopsis suae	CGMCC 3.23546	*Rhododendron delavayi*	China	OP082428	OP185514	OP185521
Pestalotiopsis taxicola	CFCC59976	*Taxus chinensis*	China	OQ626673	OQ714338	OQ714333
Pestalotiopsis taxicola	CFCC59978	*Taxus chinensis*	China	OQ771893	OQ779480	OQ779485
Pestalotiopsis telopeae	CBS 114137	*Protea* sp.	Australia	KM199301	KM199559	KM199469
Pestalotiopsis telopeae	CBS 114161 T	*Telopea* sp.	Australia	KM199296	KM199500	KM199403
Pestalotiopsis telopeae	CBS 113606	*Telopea* sp.	Australia	KM199295	KM199498	KM199402
Pestalotiopsis terricola	CBS 141.69 T	soil	Pacific Islands	MH554004	MH554438	MH554680
Pestalotiopsis thailandica	MFLUCC 17-1616 T	*Rhizophora mucronata*	Thailand	MK764285	MK764329	MK764351

Pestalotiopsis trachicarpicola	OP068 T	*Trachycarpus fortunei*	China	JQ845947	JQ845946	JQ845945
Pestalotiopsis trachicarpicola	IFRDCC 2403	*Podocarpus macrophyllus*	China	KC537809	KC537816	KC537823
Pestalotiopsis trachicarpicola	LC4523	*Camellia sinensis*	China	KX895011	KX895230	KX895344
Pestalotiopsis tumida	CFCC 55158 T	*Rosa chinensis*	China	OK560610	OL814524	OM158174
Pestalotiopsis unicolor	MFLUCC 12-0276 T	*Rhododendron* sp.	China	JX398999	–	JX399030
Pestalotiopsis unicolor	MFLUCC 12-0275	unidentified tree	China	JX398998	JX399063	JX399029
Pestalotiopsis verruculosa	MFLUCC 12-0274 T	*Rhododendron* sp.	China	JX398996	JX399061	–
Pestalotiopsis yanglingensis	LC4553 T	*Camellia sinensis*	China	KX895012	KX895231	KX895345
Pestalotiopsis yanglingensis	LC3412	*Camellia sinensis*	China	KX894980	KX895197	KX895312
Pestalotiopsis yunnanensis	HMAS 96359 T	*Podocarpus macrophyllus*	China	AY373375	–	–
Pestalotiopsis zhaoqingensis	ZHKUCC 23-0825	Unkonwed	China	OR233336	OR239061	OR239062
Pseudopestalotiopsis indica	CBS 459.78	*Hibiscus rosa-sinensis*	India	KM199381	KM199560	KM199470
Pseudopestalotiopsis theae	MFLUCC12-0055	*Ceriops tagal*	Thailand	JQ683727	JQ683743	JQ683711
Pseudopestalotiopsis cocos	CBS 272.29	*Cocos nucifera*	Indonesia	KM199378	KM199553	KM199467

[1] Acronyms: ATCC: American Type Culture Collecton, Virginia, USA; BBH: BIOTEC Bangkok Herbarium, National Science and Technology Development Agency, Thailand; CBS: Westerdijk Fungal Biodiversity Institute (CBS-KNAW Fungal Biodiversity Centre), Utrecht, The Netherlands; CFCC: China Forestry Culture Collection Centre, Beijing, China; CMW: Culture collection of Michael Wingfield, University of Pretoria, South Africa; CPC: Culture collection of Pedro Crous, The Netherlands; IMI: Culture collection of the International Mycological Institute, CABI Bioscience, Egham, Surrey, UK; MFLU: Mae Fah Luang University herbarium, Thailand; MFLUCC: Mae Fah Luang University Culture Collection, Thailand; MUCC: Murdoch University Culture Collection, Perth, Australia; NE: Gerard Adams collections, University of Nebraska, Lincoln NE, USA; PPRI: Culture collection of the Plant Protection Research Institute, Agriculture Research Center, Pretoria, South Africa; XJAU: Xinjiang Agricultural University, Xinjiang, China; NA: not applicable. All the new isolates used in this study are in bold and the type materials are marked with T.
